# The Protein Engineering of Zearalenone Hydrolase Results in a Shift in the pH Optimum of the Relative Activity of the Enzyme

**DOI:** 10.3390/toxins16120540

**Published:** 2024-12-13

**Authors:** Anna Dotsenko, Igor Sinelnikov, Ivan Zorov, Yury Denisenko, Aleksandra Rozhkova, Larisa Shcherbakova

**Affiliations:** 1 Federal Research Centre “Fundamentals of Biotechnology” of the Russian Academy of Sciences (RAS), 119071 Moscow, Russia; a.dotsenko@fbras.ru (A.D.); sinelnikov.i@list.ru (I.S.); denisenkoyura@mail.ru (Y.D.); 2 All-Russian Research Institute of Phytopathology of RAS, Bolshie Vyazemy, 143050 Moscow, Russia; 3 Department of Chemistry, Lomonosov Moscow State University, 119991 Moscow, Russia; inzorov@mail.ru

**Keywords:** zearalenone, enzymatic degradation, lactonohydrolase, recombinant proteins, protein engineering, pH profile

## Abstract

An acidic shift in the pH profile of *Clonostachys rosea* zearalenone hydrolase (ZHD), the most effective and well-studied zearalenone-specific lactone hydrolase, is required to extend the range of applications for the enzyme as a decontamination agent in the feed and food production industries. Amino acid substitutions were engineered in the active center of the enzyme to decrease the pKa values of the catalytic residues E126 and H242. The T216K substitution provided a shift in the pH optimum by one unit to the acidic region, accompanied by a notable expansion in the pH profile under acidic conditions. The engineered enzyme demonstrated enhanced activity within the pH range of 3–5 and improved the activity within the pH ranging from 6 to 10. The D31N and D31A substitutions also resulted in a two-unit shift in the pH optimum towards acidic conditions, although this was accompanied by a significant reduction in the enzyme activity. The D31S substitution resulted in a shift in the pH profile towards the alkaline region. The alterations in the enzyme properties observed following the T216K substitution were consistent with the conditions required for the ZHD application as decontamination enzymes at acidic pH values (from 3.0 to 6.0).

## 1. Introduction

Along with other toxins produced by *Fusarium* fungi, zearalenone (ZEA) is a world-wide contaminant of feeds and other agricultural products [1,2,3,4]. This mycotoxin poses risks to animal and human health mainly because of its estrogenic activity [5]. The ingestion of ZEA-contaminated feeds causes various endocrine disorders in animals and critically reduces their reproductive ability [6,7], resulting in significant economic losses in livestock and poultry farming [8]. ZEA is a compound resistant to different treatments, and it still remains toxic during feed and food processing [9,10]; therefore, its efficient detoxification by chemical or physical methods requires conditions, which often result in a significant decline in the quality of feed and other agricultural commodities. At the same time, the use of enzymatic preparations to detoxify ZEA represents a promising environmentally safe approach preserving the nutritional value of feeds and food products [11]. It is well documented that the enzymes belonging to lactonases, peroxidases, laccases, and CoA thioesterases and that are produced by some bacteria, actinomycetes, yeasts or filamentous fungi, are able to destroy this mycotoxin with the production of less toxic non-estrogenic metabolites [12]. For instance, lactonases, which cleave ZEA lactone ring and induce further spontaneous degradation of the hydrolyzed toxin derivative, were isolated from *Aeromicrobium* sp., *Clonostachys rosea*, *Cladophialophora bantiana*, *Exophiala aquamarina*, *E. spinifera*, *Phialophora americana*, *P. attinorum*, *Rhodococcus erythropolis*, and *Trichoderma aggressivum* [13,14,15,16,17,18]. Enzymatic ZEA degradation in grain feeds can be a practical and economical method for their decontamination [13,19]. Thus, the potential for ZEA detoxification in the animal gastrointestinal tract by commercial preparation ZENzyme^®^ (BIOMIN Holding GmbH, Getzersdorf, Austria) based on zearalenone hydrolase from *R. erythropolis* PFA D8-1 has been recently reported [20].

One of the most effective and well-studied ZEA-specific lactone hydrolases (ZHDs) of *C. rosea*, successfully expressed in pro- and eukaryotic organisms, was shown to provide rapid toxin degradation in model solutions, contaminated forage grain [21,22], and other products [23] under controlled conditions. With regard to its high target efficacy and the availability of easily cultivated recombinant producers, ZHD from *C. rosea* looks promising for industrial use. However, the optimal pH values of this ZHD are 8.3–8.5 [13,22,24]. Moreover, other ZEA-specific lactonases are characterized by even higher alkaline optimum pH values ranging from 9 to 10 [15]. Such a property can limit the scope of ZHDs’ application as decontamination enzymes for feed treatment, since the pH values, preventing the development of pathogenic bacteria in forage feeds (e.g., in silage and cereal- and grain legumes-based feeds) and promoting better feed digestion in the cows’ and goats’ rumens, are in the acidic zone (from 3.8 to 6.0) [25,26]. In the case of application as a putative decontamination feed additive for monogastric animals (e.g., pigs or poultry), these enzymes should be active under more acidic conditions ranging from 3 to 4 [19,27,28].

One way to improve the ZHD applicability in feed production is protein engineering aimed at obtaining enzyme variants with desirable properties. The prospects of the rational design of ZHD were recently shown for enhancing its thermostability [17] as well as its hydrolytic activity towards α-zearalanol, a highly toxic ZEA derivative [29]. In addition, an engineered variant of *C. rosea* zearalenone lactonase ZHD101 was found to possess an increased ZEA-degrading activity under acidic conditions, improving thermostabilty and effectively degrading the ZEA in a pig stomach chyme [19]. Substitutions to the residues K and D on the protein surface were reported to increase the enzyme activity of *C. rosea* IFO 7063 zearalenone lactonase ZHD101 under the acidic pH values 4.2–6 while maintaining the same pH optimum [19,30]. However, protein engineering methods have not been previously used to shift the pH optimum of ZHD.

Nevertheless, various protein engineering approaches have been used for other hydrolases [31,32,33,34,35]. For example, the D117N single-point mutation in xylanase B of *Aspergillus niger* resulted in a pH shift of 0.5 units compared to the wild-type enzyme [32], and a deletion of the proline-rich C-terminus of xylanase A isolated from sheep rumen resulted in an extension of its pH optimum, thus providing increased activity of the enzyme under the physiological conditions of the rumen (pH 5.0–7.0, 39 °C) [31]. In another study [33], a substitution E228K was introduced into phytase A originating from *A. niger* that shifted the pH optimum from 5.5 to 3.5 compared to the wild-type enzyme.

The relationship between the structures and activities of the engineered enzyme variants was analyzed by modeling the structures of different variants of endoglucanase II from *Trichoderma reesei* [34]. According to the obtained results, sequential saturation mutagenesis at the N342 position resulted in a shift in the pH optimum to the alkaline side with a simultaneous improvement in the catalytic activity of the enzyme. The introduction of a single-point mutation at the D98 position in the endoglucanase III of *Penicillium verruculosum* resulted in a shift in the enzyme pH optimum from pH 4.0 (wild-type enzyme) to 5.1 [35].

In the current study, we applied protein engineering technology to shift the pH optimum of recombinant ZHD to a more acidic area. The structure of the mutant complex of *C. rosea* ZHD with ZEA (PDB ID 3WZM) was used as a template for the docking optimization.

## 2. Results

### 2.1. Analysis of Tertiary Structure of Zearalenone Hydrolase

**Figure 1:**
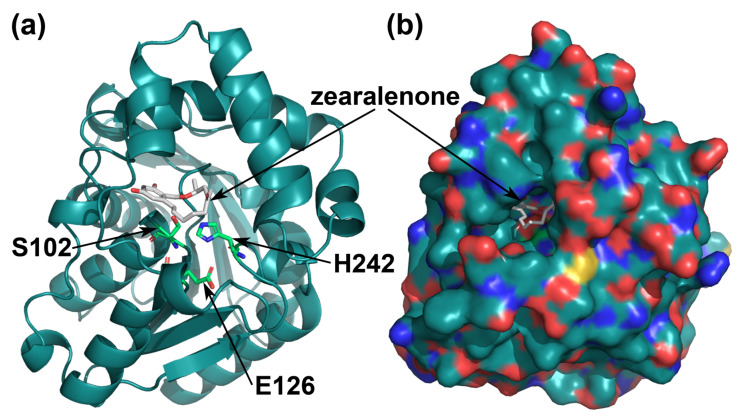
The tertiary structure of *C. rosea* ZHD. The S102-H242-E126 catalytic triad in the active center of the enzyme; the residues S102, H242, and E126 are shown in green (**a**); (**b**) the pocket of the active center. The molecule of the substrate (zearalenone) is shown in gray; atoms of nitrogen, oxygen, and sulfur in the structure of the enzyme are shown in blue, red, and yellow, respectively.

The active center of the enzyme included the S102-H242-E126 catalytic triad (Figure 1a) [36] and had the form of a pocket inside of the protein globule (Figure 1b). Amino acid substitutions that are located near the catalytic triad can affect enzyme activity and generate a shift in a pH profile. In the active center of *C. rosea* ZHD, amino acid residues S103, G213, T216, and F221 were located within 3.5 Å from the residues of the catalytic triad. These positions (103, 213, 216, and 221) were theoretically substituted with 19 other amino acid residues. The values of pKa were calculated for the catalytic residues H246 and E126, while the residue S102 from the catalytic triad was considered not titratable. Since the pH optimum of enzyme activity depends on the values of the pKa of the catalytic residues, the selection was aimed at those substitutions providing a decrease in the pKa values to shift the optimum from the alkaline area to the acidic one.

Substitutions at the 103 position have no considerable effect on pKa values (Table S1). In the case of the other three positions, different substitutions generated either a decrease in the pKa values or an increase (Table S1 for G213, Table S2 for T216 and F221). The substitutions having a decreasing or neutral effect on the pKa values are listed in Table 1.

A mechanism of ZEA degradation was described for *Rhinocladiella mackenziei* zearalenone hydrolase previously [37]. In particular, the amino acid residues D34 and H128 in *R. mackenziei* zearalenone hydrolase were reported to slow the reaction because, due to their electronic influence, they make the reactant more stable than the transition state in the reaction mechanism [37]. The sequence identity of *R. mackenziei* zearalenone hydrolase and the enzyme in this study is 64.3%. At the same time, tertiary structures of the enzymes have a similar fold with RMSD of 0.4 Å. The residues D34 and H128 in *R. mackenziei* zearalenone hydrolase are equal to D31 and H125 in the enzyme in this study. Therefore, both residues, D31 and H125, were also considered for mutagenesis.

In the case of the residue D31, no substitutions affected the pKa value of the catalytic residue E126. However, several substitutions affected the pKa of the catalytic residue H242 (Table S3). The maximum decrease was calculated for the substitutions with the R and K amino acid residues; the effect was similar to the effect of the substitutions with the R and K amino acid residues at positions G213, T216, and F221 (Table 1). However, the substitutions D31R and D31K resulted in destabilization of the tertiary structure of the enzyme due to the high values of ΔΔG. The substitutions D31F, D31W, and D31Y provided a decrease of –0.66 to –0.74 in pKa and were neutral for the stability, according to the low values of ΔΔG (Table 1). Substitutions at the H125 position had no considerable effect on the pKa value of the catalytic residue H242, but they greatly increased the pKa value of the catalytic residue E126 (Table S3).

**Figure 2:**
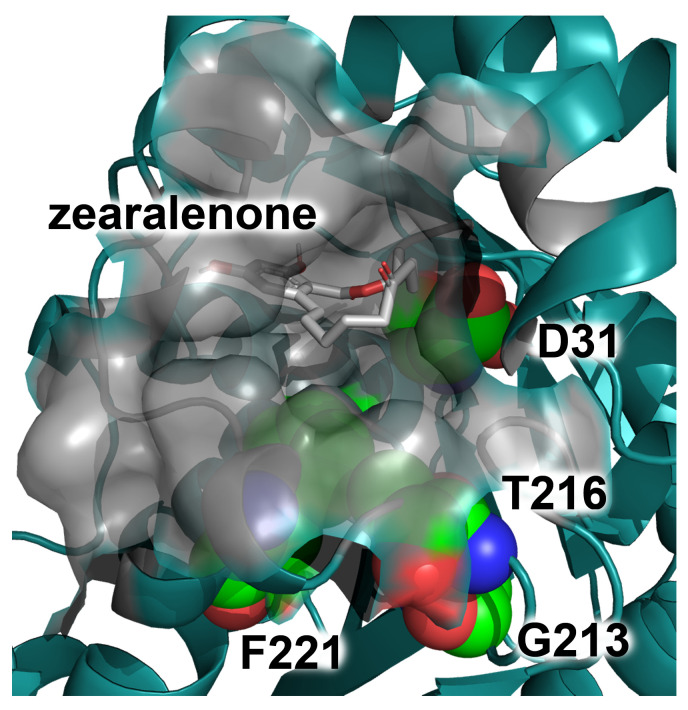
Location of the residues G213, T216, and F221 near the entrance to the pocket and the residue D31 at the bottom of the active center pocket of *C. rosea* ZHD. The substrate molecule (zearalenone) is shown in gray; the surface of the pocket of the active center is shown in transparent gray; the residues D31, G213, T216, and F221 are shown in green spheres, with nitrogen and oxygen atoms shown in blue and red, respectively.

The positions D31, G213, T216, and F221 presented in Table 1 differ by their location in the pocket of the active center. The positions G213, T216, and F221 were located near the entrance to the pocket, close to each other, while the position D31 was at the bottom of the pocket (Figure 2). The substitutions G213 to R or K decreased the pKa values and had a minor effect on the enzyme affinity to ZEA and also on the surface area and volume of the pocket of the active center. The substitutions G213 to S or T decreased only the pKa of the residue E126, but not H242, and had no effect on the affinity and the dimensions of the pocket of the active center. In the case of the residue T216, the substitutions to R and K considerably decreased the values of pKa for both catalytic residues, with a minor improvement in the affinity and a narrowing of the pocket of the active center. The substitution T216H, if compared to T216R and T216K, caused lower changes in the affinity and dimensions of the pocket (Table 1).

The substitutions at the positions F221 and D31 (Table 1) were characterized with high values of ΔΔG, meaning the destabilization of the tertiary structure of the enzyme. Also, all these substitutions, except for D31K, caused a reduction in the affinity. The substitutions D31H and F221H were considered previously for charge redesign in the pocket of the active center of *C. rosea* IFO 7063 zearalenone lactonase ZHD101. The variants D31H and F221H were predicted to have largely increased the free energy of folding and largely increased the binding energy of the substrate ZEA, respectively, and the variants were not evaluated experimentally [30].

**Figure 3:**
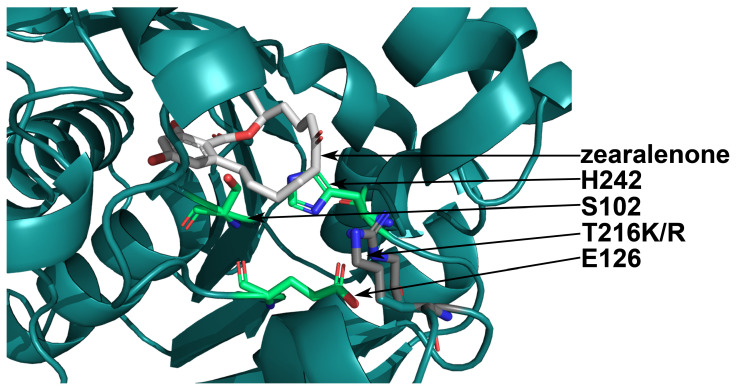
The tertiary structure of *C. rosea* ZHD with the T216K and T216R amino acid substitutions. The S102-H242-E126 catalytic triad in the active center of the enzyme is shown in green, the substrate molecule (zearalenone) is shown in gray, and the T216K and T216R substitutions are shown in dark gray. Nitrogen and oxygen atoms are shown in blue and red, respectively.

Of the substitutions listed in Table 1, only G213R, T216R, and T216K were calculated to decrease the pKa of both the catalytic residues E126 and H242, improve or have a minor effect on the affinity, and have a minor effect on ΔΔG. According to multiple sequence alignment (MSA), the residues D31, G213, T216, and F221 were characterized with 65, 100, 69, and 91% frequency, respectively, with G213 and T216 being conservative and non-conservative, respectively. Two other positions, S103 and H125, for which no decrease in the pKa values was predicted, were characterized with high frequency in MSA—98 and 100%, respectively. It is worth mentioning that the substitutions T216R and T216K were similar in the predicted effect on such parameters as pKa, ΔΔG, the affinity, and the dimensions of the pocket of the active center. While the substitution T216K was predicted with CUPSAT [38] as 2.53 kcal/mol more preferred over T216R according to the overall calculations of atom potentials and torsion angle potentials. Additionally, the K residue was more compact than R, and the site of mutagenesis T216 was located in close proximity to the entrance to the active center pocket (Figure 3).

**Figure 4:**
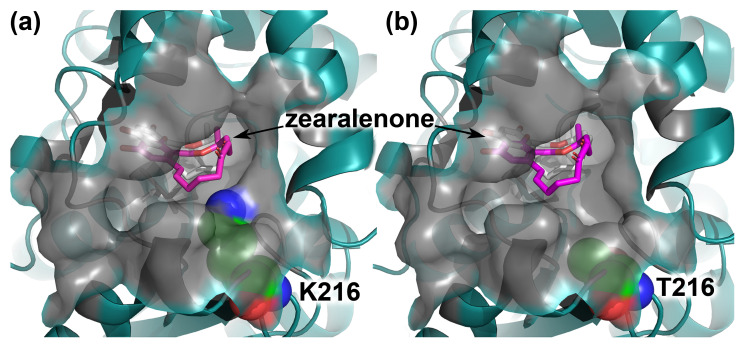
The tertiary structure of *C. rosea* ZHD with the T216K amino acid substitution (**a**) and the wild-type enzyme (**b**). The surface of the active center pocket is shown in transparent gray; the molecule of the substrate zearalenone is shown in gray and magenta for the modes at the bottom of the pocket and closer to the entrance to the pocket, respectively; the residues K216 and T216 are shown in green spheres, with nitrogen and oxygen atoms shown in blue and red, respectively.

According to the docking of ZEA in the active center, the variant with the substitution T216K differed from the wild-type enzyme, not only in the value of the affinity, but also in the presence of the secondary sites of binding. In the variant T216K, the best mode with the affinity −9.0 kcal/mol corresponded to the location of ZEA close to that in the complex of mutant *C. rosea* zearalenone hydrolase S102A with ZEA (PDB ID 3WZM). The sequence identity of the enzyme with PDB ID 3WZM and the enzyme ZHD in this study was 98.9%. The high degree of sequence identity allowed the comparison of the location of ZEA in the active centers of these enzymes. The less preferred mode had the affinity of −7.3 kcal/mol and defined the location of ZEA remote from the catalytic residues and closer to the entrance to the pocket of the active center (Figure 4a). In the wild-type enzyme, the mode equal to that in the crystal structure had the affinity −8.8 kcal/mol and was less preferred than the mode with ZEA located closer to the entrance (−9.0 kcal/mol) (Figure 4b). The pocket of the active center had a narrower entrance in the case of the variant T216K if compared to the wild-type enzyme (Figure 4). Also, in the case of the variant T216K, the pocket was characterized with decreased surface area and volume (Table 1). The changed dimensions of the pocket made the binding of ZEA at the bottom of the active center more preferred than a remote mode near the entrance to the pocket. The mentioned difference between the variant T216K and wild type was determined not only at pH 8.0 (Figure 4), which is the optimal pH for the activity of *C. rosea* ZHD, but also in a wide range of pH values from 3.0 to 10.0 (Table S4).

**Figure 5:**
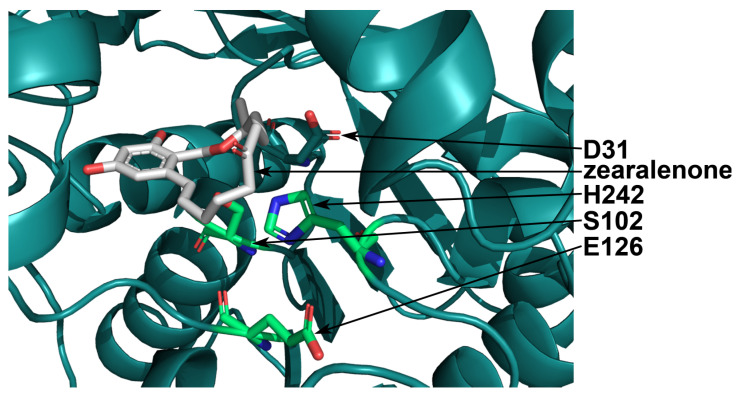
The tertiary structure of *C. rosea* ZHD with the residue D31 at the bottom of the active center pocket. The S102-H242-E126 catalytic triad in the active center of the enzyme is shown in green, and the molecule of the substrate zearalenone is shown in gray. Nitrogen and oxygen atoms are shown in blue and red, respectively.

The special role of the residue D31 in the catalysis was reported previously. In *R. mackenziei* zearalenone hydrolase, the equal residue D34 was supposed to slow the reaction because, due to its electronic influence, it makes the reactant state more stable than the transition one [37]. Also, the residue D31 was supposed to interact with the catalytic residue H242, similarly to the residue E126, and fix the imidazole ring of H242 during substrate binding [39]. The residue D31 was located close to the residue H242 in the active center of *C. rosea* ZHD (Figure 5) and at the bottom of the pocket of the active center (Figure 2). The substitutions of D31 with R, K, F, W, and Y provided a decrease in the pKa value of the residue H242; however, this caused destabilization of the tertiary structure and reduction in the affinity (Table 1).

Therefore, for the residue D31, no substitutions were designed to shift the pH optimum of the activity of the enzyme. However, several substitutions could be considered to test the effect of the residue D31 on the enzyme activity, since such an effect was supposed previously [37,39]. The N residue is quite similar to D from the steric point of view but differs in the electronic influence. Two more residues, A and S, were also considered for the site-directed mutagenesis. The residue A was selected as the most compact aliphatic amino acid residue but not flexible G. The S residue was selected by MSA since its frequency was 35% (in addition to the D residue characterized by the 65% frequency).

### 2.2. The Production of Mutant Forms of Zearalenone Hydrolase, Their Isolation, and Subsequent Identification

**Figure 6:**
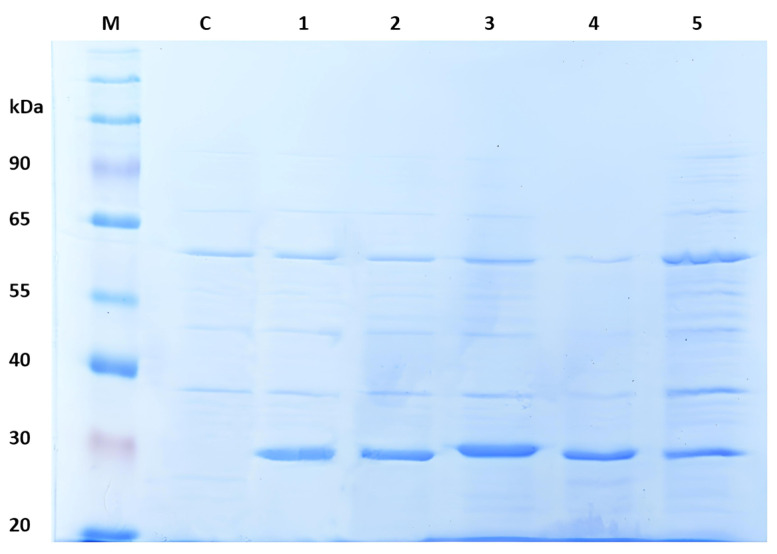
SDS-PAGE electropherogram of the crude lysates of *E. coli* cells (C—non-induced control; 1—native ZHD; 2—ZHD(T216K); 3—ZHD(D31A); 4—ZHD(D31S); 5—ZHD(D31N) and M—molecular weight marker).

The *E. coli* Arctic Express strain was used to cultivate the cells that had been transformed with pNIC-ZHD plasmids encoding the native ZHD protein or one of four mutant variants (T216K, D31A, D31S, and D31N) of this protein. The cultivation medium was Terrific Broth supplemented with 50 μg/mL kanamycin. Following induction with isopropyl-β-D-thiogalactopyranoside (IPTG), the cultures were harvested and analyzed by SDS-PAGE (12.5% acrylamide/bisacrylamide), carried out under reducing conditions. Figure 6 displays a Coomassie-stained SDS-PAGE gel with recombinant ZHDs (rZHDs), with an approximate molecular weight of 30 kDa. Following the disruption of the bacterial cells by ultrasonication, the rZHDs were purified from the soluble fraction of the cell lysates using immobilized metal affinity chromatography (IMAC). The purification of the enzymes was achieved using a one-step chromatographic process that was consistent for all the rZHDs (see Section 4.4 for details).

### 2.3. The Effect of the Introduced Mutations on the pH Optimum of ZHD

**Figure 7:**
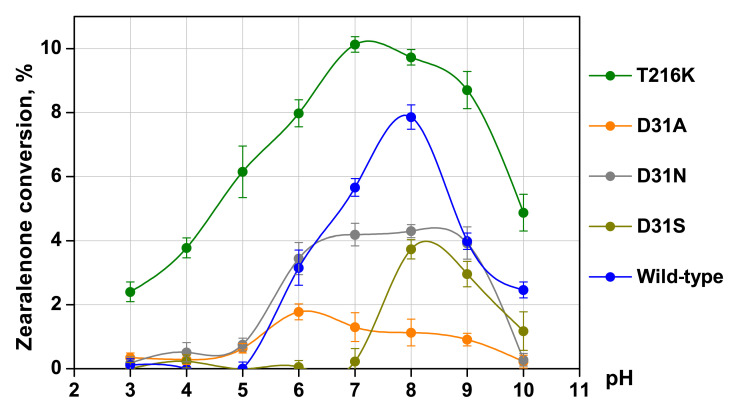
The influence of pH on the zearalenone conversion by mutant and wild-type forms of *C. rosea* ZHD. Y-bars on curves show standard deviation (*p* < 0.05).

The HPLC determination of zearalenone conversion by the native ZDH and four mutant variants was performed at pH values ranging from 3 to 10. A ZEA solution (5 μg/mL) dissolved in an appropriate buffer was treated with ZDH. Samples were taken from the reaction mixture, diluted with acetonitrile, centrifuged, and analyzed by HPLC. The change in the ZEA concentration in the reaction mixture was determined by measuring the absorbance at 236 nm (Figure 7).

The wild-type enzyme was found to exhibit the maximum activity at pH 8.0, with the enzyme retained activity between pH 6.0 and 10.0 (Figure 7). The variant T216K demonstrated an extended pH profile, with the activity maximum shifted by approximately one unit to the acidic region compared to the wild type. At the pH optimum 7.0, the variant T216K showed 1.3 times higher activity than the wild-type enzyme at pH 8.0, with the residual activity observed in the acidic pH range up to pH 3.0. The variant T216K retained 24–61% of its maximal activity at pH 3.0–5.0, while the enzyme of the wild type was inactive at these pH values.

Compared to the wild-type enzyme, the activity of the variant D31A was 1.8, 4.4, and 7.0 times lower at pH values 6.0, 7.0 and 8.0, respectively. The variant D31A displayed no activity at pH 10.0, when the enzyme of the wild type retained 32% of its maximal activity. At the same time, the variant D31A exhibited an evident shift in the pH optimum towards acidic pH values, from pH 8.0 for the wild-type enzyme to pH 6.0, than that observed for the variant T216K.

The variant D31N did not show a notable alteration in its pH profile when compared to the WT enzyme, except for the alkaline region (within 9.0–10.0), where the activity of this variant was declined. The variant D31N was inactive at pH 10.0 similarly to the variant D31A. At pH values 6.0 and 9.0, the variant D31N displayed activity similar to the WT enzyme. At pH values 7.0 and 8.0, close to pH optima for both the variant D31N and the WT enzyme, the activity of this variant was decreased by 1.4–1.8 times.

The variant D31S demonstrated a shift in its pH optimum to a more alkaline region. The variant D31S showed no ZEA-degrading activity at pH values below 7.0, and its activity at the optimum pH was twice as low as that of the wild-type enzyme.

ZEA-degrading enzymes are generally not acidic enzymes. Optimal pH values were reported in a range of 7.0–9.5 for the ZEA-degrading enzymes from *Gliocladium roseum*, *C. bantiana*, *E. aquamarina*, *Neurospora crassa*, *P. americana*, *R. mackenziei*, and *C. rosea* [14,19]. However, the ZEA-degrading enzymes from *C. bantiana*, *N. crassa*, and *C. rosea* were also reported to remain active at pH 4.2 [19]. The substitution K94D located on the protein surface of *C. rosea* zearalenone lactonase ZHD101 was reported to increase the hydrolysis efficiency by 1.4–3.8 times at pH 3.8–5.0 [19]. Substitutions with K on the protein surface of *C. rosea* IFO 7063 ZHD101 were shown to enhance the catalytic efficiency up to 1.3 times at pH 5.5 [30]. A comparison of these reports confirms that the improvement in the activity obtained in the present study for the variant T216K at pH 3.0–5.0 can be considered a good result of the rational design of the enzyme active center for shifting the pH profile.

Among the performed substitutions, the substitution T216K resulted in the most pronounced and beneficial changes in the activity of *C. rosea* ZHD. In addition to the increase in the activity at pH 6.0–10.0, the range where the wild-type enzyme demonstrated the main activity, the variant T216K had a minor activity at pH 3.0–5.0, the range where no activity was detected for the wild-type enzyme (Figure 7). According to the calculations of the pKa values, the substitution T216K caused a decrease in the pKa value of the catalytic residue H242 from 4.65 to 3.65, the residue providing a proton transfer in the supposed mechanism of ZEA degradation [37]. Also, the substitution T216K improved the binding of the substrate molecule ZEA in the active center pocket in a pH range of 3.0–10.0, according to the docking simulations (Figure 4, Table 1 and Table S4).

The wild-type enzyme and all the variants were predicted to have an equal tendency for aggregation and similar solubility in a wide range of pH. The method for the aggregation predictions exploits lipophilicity and pKa values of amino acid residues with structural corrections [40], while the solubility predictions use calculations of partial charges, hydrophobicity values, and pKa values [41]. The solubility cannot be, in general, assumed as the inverse of aggregation propensities; therefore, both characteristics were valued.

In the case of aggregation, the wild-type enzyme and all the variants had almost constant aggregation propensities in a pH range of 6.0–9.0 with worsening in a range of 4.0–5.0 (Figure S1). The tool applied for the aggregation prediction, Aggrescan4D, allows predictions only in the range of 4.0–9.0, but not at pH 3.0 and 10.0. In the case of solubility, the wild-type enzyme and the variants D31A, D31S, and D31N had similar solubility in a pH range of 3.0–5.0, while the variant T216K had a slightly improved solubility under these values of pH (Figure S2). In a pH range of 6.0–10.0, the variant T216K was predicted to be more soluble than the wild-type enzyme, and the variants D31A, D31S, and D31N were predicted to be less soluble than the wild-type enzyme (Figure S2).

The structure flexibility of the variant T216K, the variant demonstrating the improvement in the enzyme activity of ZHD, was compared to that of the wild-type enzyme. The values of the root mean square fluctuation of the amino acid residues were similar for the wild-type enzyme and the variant T216K, with a minor improvement for a set of residues (Figure S3). According to the predictions of solubility, aggregation propensities, and structure flexibility, the substitution T216K had a minor effect on the entire tertiary structure of the enzyme. Meanwhile, the obtained improvement in the enzyme activity correlated with the decrease in the pKa value of the catalytic residue H242 and the improvement in the binding of the substrate molecule ZEA in the active center pocket.

## 3. Discussion

The substitution T216K resulted in a shift in the pH optimum of the enzyme by one unit to the acidic region along with an increase in the width of the pH profile of its activity (Figure 7). According to the supposed mechanism of ZEA degradation, the reaction is started with a proton transfer from S to H in the S-H-E catalytic triad, followed by a nucleophilic attack of the oxygen atom of the residue S to the carbonyl carbon of the substrate molecule, resulting in a cleavage of the substrate [37]. The pKa value of the catalytic residue H242 was calculated to decrease from 4.65 for the wild-type enzyme to 3.65 for the variant T216K. The calculated shift correlated with the shift in the pH profiles of activity. In the pH profiles, the wild-type enzyme demonstrated no activity below pH 5.0, while the variant T216K retained 23–38% of its maximal activity at pH 3–4 (Figure 7).

The overall increase in the enzyme activity within a wide pH range can be explained by the increased acidic properties of the residue H242 and an optimized conformation of the pocket of the active center. As mentioned above, for the supposed mechanism of ZEA degradation, a proton transfer from S to H occurred in the S-H-E catalytic triad [37]. The deprotonation of the S residue can be improved by increasing the acidic properties of the H residue. In the case of the optimization of the conformation of the active center pocket, a similar effect was reported for *P. americana* lactone hydrolase ZHD607 [14]. *P. americana* ZHD607 shared 63% of its sequence identity with *C. rosea* zearalenone hydrolase (PDB ID 3WZM) and demonstrated the maximal activity towards ZEA at pH 8.0. With the substitution I160Y, which was located closely to the entrance of the pocket of the active center, the pocket was covered by Y160 and formed a more closed conformation, making better binding of ZEA. The enzyme activity was increased 3.4 times at pH 8.0; changes in the pH profile were not tested and reported [14]. In the case of *C. rosea* ZHD in this study, the substitution T216K narrowed the entrance to the pocket of the active center. This made the binding of ZEA at the bottom of the pocket more preferred than the unproductive binding displaced to the entrance to the pocket (Figure 4). The surface area and volume of the pocket were considerably decreased (Table 1). Also, a minor improvement in the affinity was calculated (Table 1). In the pH profiles, the variant T216K demonstrated up to a 2.5-fold increase in the activity at pH 6–10 compared to the wild type and remained active at pH 3–5 with no activity for the wild-type enzyme (Figure 7).

The D31N, D31A, and D31S substitutions resulted in the decreased activity of the enzyme in a wide pH range, with the D31N substitution having the least effect on the enzyme activity (Figure 7). The molecular structure of the N residue was similar to that of the D residue but carried a smaller negative charge. The pH optimum of the variant D31N was shifted to the acidic area compared to the wild-type enzyme. A similar shift was obtained in the case of the D31A substitution with the introduction of the aliphatic residue A in the 31 position. Also, this substitution resulted in a more dramatic decrease in the enzyme activity than the D31N substitution (Figure 7). The residue D31 not only provided an electronic influence on the catalytic residues but also affected the tertiary structure of the enzyme and the active center conformation. The high ΔΔG values in the case of the D31A substitution indicate a destabilized tertiary structure (Table S3). In the case of the D31S variant, the pH optimum was shifted to the alkaline area, and the overall activity was decreased, which can be caused by both electronic influence on the catalytic residues and the destabilization of the tertiary structure of the enzyme.

The residue D31 was reported previously to affect the stability of the reactant state in the supposed mechanism of the catalytic reaction due to its electronic influence [37]. Also, this residue was supposed to fix the imidazole ring of the catalytic residue H242 during substrate binding in the active center of zearalenone hydrolases [39]. A considerable decrease in the activity for the variants D31A and D31S revealed the importance of the residue D31 for the activity of *C. rosea* ZHD (Figure 7).

A charge redesign in the pocket of the active center was reported for *C. rosea* IFO 7063 zearalenone lactonase ZHD101. The residue H was introduced in the active center pocket to provide an additional weakly positive charge and to reduce the pKa value of the catalytic residue H242. The substitutions D31H and F221H were calculated to largely increase the free energy of the folding and binding energy of the substrate ZEA, respectively, and were not evaluated experimentally. The substitutions V158H, M154H, and V153H decreased the catalytic efficiency by 4.8–14 times at pH 5.5. At the same time, the substitutions V153H and V158H had no or minor effect on the enzyme activity at a neutral pH [30]. Substitutions with K of the residues located on the protein surface of *C. rosea* IFO 7063 ZHD101 at a distance of 8–20 Å from the catalytic residue H242 were also evaluated to increase the enzyme activity under acidic pH values. Two substitutions, D157K and E171K, of the nine tested ones increased the catalytic efficiency 1.3 times at pH 5.5 while maintaining the same pH optimum of 7.0 [30].

The methodology for the pH shift tested in the present study was successful. The substitution T216K provided a shift in the enzyme pH optimum by one unit to the acidic region and enlarged the pH profile range. The variant was active at the pH values of 3.0–10.0 while the wild-type enzyme remained active at pH values of 6.0–10.0 (Figure 7). However, the approach was limited to the range of those amino acid residues that were located in the pocket of the active center closely to the catalytic residues. In the case of *C. rosea* ZHD in this study, four residues S103, G213, T216, and F221 were detected for consideration as located within 3.5 Å from the residues of the S102-H242-E126 catalytic triad. Two more residues, D31 and H125, were also considered for the rational design due to their effect on the stability of the transition state in the reaction mechanism reported previously [37]. Of these six residues, two residues were calculated to have no decreasing effect on the pKa values of the catalytic residues, namely S103 and H125. In the case of the positions D31 and F221, the substitutions providing a decrease in the pKa values were predicted to negatively affect ΔΔG and affinity to the substrate ZEA. The position G213 was conservative with a 100% frequency in a multiple sequence alignment. The only position for the experimental evaluation was position T216. For further improvement of the enzyme properties, substitutions on the protein surface at a distance from the catalytic residues may be performed. Such studies were reported for *C. rosea* IFO 7063 ZHD101. The activity of the enzyme at the acidic values of pH was increased through substitutions to the residues K [30] and D [19] located on the protein surface.

Along with other studies, the results of our research clearly demonstrate the promise of the rational design of the ZHD active center for the extension of the pH profile and for shifting the enzyme pH optimum to the acidic area. Obtaining the ZHD variants engineered in our study, especially the enzyme mutant with the T216K substitution, which showed an increased activity within the pH range of 3–6, can be considered the first step towards the development of ZHD-based enzyme preparations for feed decontamination. Although additional investigations are necessary to confirm the stability of the T216K functioning at pH 3.0 for an allotted time, retention of the ZEA-degrading activity in the range of acidic pH values is an important property of this mutant from a practical point of view. In particular, the ZEA-degrading activity in the pH range of 3–4 is consistent with the conditions required for the ZHD application as a feed additive for livestock and poultry, while the activity at pH values from 3.8 to 6.0 is appropriate for the ZHD usage as a decontamination enzyme when feed processing.

## 4. Materials and Methods

### 4.1. Analysis of Zearlenone Hydrolase Tertiary Structure

The tertiary structure of the enzyme was obtained using a SWISS-MODEL bioinformatic tool (available at https://swissmodel.expasy.org, accessed on 8 December 2024) [42]. The structure of a zearalenone hydrolase from *Clonostachys rosea* (PDB ID 3WZM) was used as a template with the sequence identity of 98.9%. A model of the enzyme with a ZEA molecule in the active center was obtained by docking in an AutoDock Vina v. 1.2.1 program [43] as a part of the AMDock v. 1.5.2 software [44].

The structure of the complex of mutant zearalenone hydrolase from *C. rosea* with ZEA (PDB ID 3WZM) was used as a template for the docking optimization. Models of the wild-type enzyme and the variants with amino acid substitutions were used for docking a molecule of ZEA into the active center to calculate the affinity in an AutoDock Vina v. 1.2.1 program [43] as a part of the AMDock v. 1.5.2 software [44]. The search space for docking was centered on the catalytic residues S102, E126, and H242. The amino acid residues located near the catalytic residues S102, E126, and H242 in the tertiary structure of the enzyme were detected using a Swiss-PDBViewer v. 4.1.0 program [45].

The distance for selecting the nearest amino acid residues was set to 3.5 Å from the OG atom of the residue S102, the CD atom of the residue E126, or the NE2 atom of the residue H242. The impact of the amino acid substitutions on the stability of the tertiary structure was calculated using a standalone version of a MAESTRO v. 1.2.35 program [46]. Calculations of the atom potentials and torsion angle potentials were performed with a CUPSAT bioinformatic tool (available at https://cupsat.brenda-enzymes.org, accessed on 8 December 2024) [38].

The pKa values for the residues E126 and H242 in the tertiary structures of the variants obtained through theoretical mutagenesis in a PyMOL v. 2.4.0 program (PyMOL Molecular Graphics System, Schrodinger, LLC, New York, NY, USA) were calculated using a PropKa v. 2.0 software [47]. Changes in the pKa values of the catalytic residues E126 and H242 were calculated manually as the difference between the pKa of the mutant and wild-type enzymes. The residue S102 from the catalytic triad was considered not titratable.

The pocket of the active center was characterized in terms of surface area and volume with a CASTp v. 3.0 program [48]. The probe radius was set at the default value of 1.4 Å.

The aggregation propensities of *C. rosea* ZHD, the wild-type enzyme, and the variants, were predicted in their folded states at different pH values using an Aggrescan4D (A4D) bioinformatic tool (available at https://biocomp.chem.uw.edu.pl/a4d/, accessed on 8 December 2024). Through the use of the tool, the tertiary structures were energetically minimized with FoldX usage, and then the aggregation propensities were calculated with a distance of aggregation of 10 Å. The propensities were estimated in terms of the average score, which is the average value for the entire tertiary structure, and the maximal score, which accounts for highly structural aggregation-prone regions in the surface. The tool provides predictions in a range of pH from 4.0 to 9.0 with a step size of 0.5 [40].

The solubility of *C. rosea* ZHD, the wild-type enzyme and the variants, was predicted using a CamSolpH v. 3.0 bioinformatic tool. The pKa correction method PROPKA was applied based on the tertiary structure. The solubility profile was calculated in a range of pH from 3.0 to 10.0 with a step size of 0.5 [41].

The structure flexibility was predicted using a CABS-flex v. 2.0 bioinformatic tool. This tool allows the modeling of large-scale conformational transitions of protein systems. The parameters for the distance restraints generator and the simulation were preset as standard ones for the tool [49].

### 4.2. Multiple Sequence Alignment

Multiple sequence alignment was obtained using a ConSurf bioinformatic tool (available at https://consurf.tau.ac.il/consurf_index.php, accessed on 8 December 2024) [50]. The total number of homologous sequences was 150, with the maximal and minimal identity between sequences being 95 and 35%, respectively.

### 4.3. Development of Plasmids with Mutated zhd101 Gene and Protein Expression

Mutant forms of ZHD were generated by introducing codon mutations into the ZHD coding sequence in the pNIC-ZHD plasmid using the QuickChange method [51] as described previously [22]. Briefly, a PCR was performed in a 20-μL reaction volume, which included 1 ng of pNIC-ZHD plasmid as the template, 0.5 μM of partially overlapping primers (Table 2), 0.5 mM dNTPs, 1U of Phusion^®^ High-Fidelity DNA Polymerase (NEB, Ipswich, MA, USA), and 4 μL of 5× Phusion HF buffer.

The PCR was carried out under the following conditions: initial denaturation at 98 °C for 3 min; 16 cycles of denaturation at 98 °C for 25 s, annealing at 60 °C for 30 s, and extension at 72 °C for 300 s; and a final extension at 72 °C for 10 min.

To remove the parental plasmid, 10 U of the DpnI restriction enzyme (NEB, Ipswich, MA, USA) were added to the reaction mixture followed by a 1 h incubation at 37 °C to digest the parental DNA. Ten microliters of the resulting mixture were used to transform NEB Turbo competent *E. coli* cells (NEB, Ipswich, MA, USA), followed by a selection of kanamycin-resistant colonies. Plasmid DNA from the positive clones was isolated using a Plasmid Miniprep Color kit (Evrogen, Moscow, Russia) and sequenced by the Sanger method to confirm the mutations. The resulting plasmids were transformed into *E. coli* Arctic Express (DE3) cells (Agilent, Santa Clara, CA, USA) for the subsequent expression and purification of recombinant ZHDs.

### 4.4. Enzyme Expression and Purification

The plasmid DNA from the positive clones was isolated using a Plasmid Miniprep Color kit (Evrogen, Moscow, Russia) and sequenced by the Sanger method to confirm the mutations. The plasmids were transformed into *E. coli* Arctic Express (DE3). The transformed *E. coli* Arctic Express (DE3) cells were cultured in Terrific Broth (TB) medium at 37 °C until an OD600 of 0.5 was reached. The expression of the recombinant proteins was induced by the addition of 0.2 mM IPTG followed by growing the bacterial cells at 15 °C for 24 h. The cells were separated by centrifugation at 5500 rpm for 10 min and resuspended in a lytic buffer (20 mM potassium phosphate buffer, pH 7.4 containing 500 mM NaCl, 20 mM imidazole, 1 mM β-mercaptoethanol, and 10% glycerol). The resulting suspension was sonicated and centrifuged at 10,000× *g* for 30 min at 4 °C. The recombinant ZHD forms were purified from the culture supernatants by IMAC to obtain homogeneous enzyme samples. The supernatants were applied to a 15 mL Ni-Sepharose Excel column (Cytiva, Marlborough, MA, USA) at a flow rate of 2 mL/min. The column was equilibrated with 20 mM potassium phosphate (K-PO_4_) buffer, pH 7.4 with 500 mM NaCl. To remove non-specifically bound proteins, the column was washed with a washing buffer (20 mM K-PO4 buffer, pH 7.4 containing 500 mM NaCl and 20 mM imidazole) at a flow rate of 2.5 mL/min. The recombinant ZHD forms were eluted in a single step with 20 mM K-PO_4_ buffer, pH 7.4, supplemented with 500 mM NaCl, and 400 mM imidazole at a flow rate of 2 mL/min. The fractions exhibiting the highest enzyme concentration were combined and dialyzed against 10 mM K-PO_4_ buffer, pH 6.0.

### 4.5. The Evaluation of the Efficacy of Zearalenone Removal from Model Solutions at Varying pH Levels

Zearalenone (Evrika, Moscow, Russia) was used as a substrate to quantify the activity of recombinant forms of zearalenone hydrolase. First, 10 μL of zearalenone (5 μg/mL) was combined with 10 μL of Britton-Robinson buffer (125 mM); the pH values ranged from 3 to 10 with a step of 1 unit. The reaction mixture was pre-heated under stirring for 5 min at 30 °C. Then, 5 μL of purified enzyme (20 μg/mL) was added to the reaction mixture. In the case of the control sample, 5 μL of the appropriate buffer was added instead of 5 μL of enzyme solution. The reaction was performed for 30 min at 30 °C. The enzymatic reaction was terminated by the addition of 5 μL of 1 M HCl to the reaction mixture. The mixture was centrifuged (10 min, 15,300 rpm), and the obtained supernatant was used for analysis. The residual ZEA concentration was determined by HPLC. The conversion of ZEA was determined as the ratio of the decrease in the concentration of ZEA to the initial one.

### 4.6. HPLC Determination of Zearalenone

The residual ZEA content in the model solutions was determined using an Agilent 1200 HPLC/FLD/DAD system (Agilent Inc., Waldbronn, Germany) equipped with a binary pump and autosampler on the Kromasil Eternity-5-C18 (250 × 4.6 mm, 5 μm) analytical column (Nouryon BV, Göteborg, Sweden) attached to a Security Guard C18 cartridge (4 × 3 mm) (Phenomenex, Torrance, CA, USA). Prior to HPLC-analyses, the samples were mixed with a solution consisting of 60% ACN and 40% 100 mM acetic acid buffer (pH 4.0) at a 1:1 ratio, and then centrifuged at 12,000 rpm for 5 min. The acetonitrile (ACN):H_2_O mobile phase was delivered at a flow rate of 1.0 mL/min, with an ACN gradient from 45% to 65% over a period of 20 min. The column temperature was maintained at 40 °C. The excitation and emission wavelengths of the fluorometric detector (FLD) were set at 275 nm and 450 nm, respectively. ZEA was determined at 236 nm using the diode-array detector (DAD).

### 4.7. Statistical Analysis

The activities of wild-type ZHDs and among the enzyme mutants in tests on the ZEN degradation in the model solutions included three replications per treatment in each experiment. Quantitative results were analyzed with STATISTICA v. 6.1 software (StatSoft Inc., Tulsa, OK, USA) and presented as the mean values of the measurements obtained in at least three independent experiments. The significance of the differences (*p* < 0.05) was determined using Student’s *t*-test for independent variables.

## Figures and Tables

**Table 1:** Effect of amino acid substitutions (aa subst.) at the positions G213, T216, F221, and D31 on the stability of the tertiary structure (ΔΔG); pKa of the catalytic residues H242 and E126; affinity to the ZEA molecule; surface area and volume of the active center pocket of ZHD from *C. rosea*.

aa Subst.	ΔΔG, kcal/mol	ΔpKa (H242)	ΔpKa (E126)	ΔAffinity ^a^, kcal/mol	ΔArea ^b^, Å^2^	ΔVolume ^b^, Å^3^
G213R	–0.50762	–2.19	–2.71	–0.1	–13.085	–3.791
G213K	–0.12674	–0.28	–1.63	–0.1	–10.122	–2.698
G213S	–0.31105	–0.02	–0.64	0	0	0
G213T	–0.43384	–0.03	–0.56	0	0	0
T216R	0.12562	–1.58	–1.46	–0.3	–41.296	–26.898
T216H	–0.02002	–0.10	–1.36	–0.1	–19.964	–7.232
T216K	0.32774	–1.00	–0.52	–0.2	–37.056	–24.895
F221R	1.79928	–1.13	–0.99	0.5	–26.591	–12.602
F221H	1.89911	–0.53	–3.85	0.2	6.923	10.202
F221K	2.27054	–1.12	–1.45	0.2	–4.262	–0.715
D31R	1.41119	–1.57	0.03	1.5	–24.687	–14.938
D31K	2.03902	–2.90	0.01	–0.1	–34.604	–24.948
D31F	0.21993	–0.66	0.03	1.7	–40.047	–25.082
D31W	0.40204	–0.69	0.06	1.8	–43.202	–24.052
D31Y	0.36048	–0.74	0.04	2.2	–49.251	–36.635

^a^ Docking at pH 8.0. ^b^ For the active center pocket.

**Table 2:** Oligonucleotides used for PCR.

Primer	Sequences 5′-3′
ZHD31A_F	ctcgttcccgctggcctcggagaat
ZHD31A_R	ctccgaggccagcgggaacgaggacaac
ZHD31N_F	gttgtcctcgttcccaatggcctcggagaat
ZHD31N_R	gccattgggaacgaggacaacgtcgggtc
ZHD31S_F	ctcgttcccagcggcctcggagaatgcc
ZHD31S_R	ctccgaggccgctgggaacgaggacaacg
ZHT216K_F	ggcgctgcgaagccaaccgagtctt
ZHT216K_R	aagactcggttggcttcgcagcgcc

## Data Availability

The original contributions presented in this study are included in the article/Supplementary Materials. Further inquiries can be directed to the corresponding authors.

## Supplementary Materials

The following supporting information can be downloaded at https://www.mdpi.com/article/10.3390/toxins16120540/s1, Table S1: Effect of amino acid (aa) substitutions at the positions S103 and G213 on the stability of the tertiary structure (ΔΔG) and pKa of the catalytic residues H242 and E126. Table S2: Effect of amino acid (aa) substitutions at the positions T216 and F221 on the stability of the tertiary structure (ΔΔG) and pKa of the catalytic residues H242 and E126. Table S3: Effect of amino acid (aa) substitutions at the positions D31 and H125 on the stability of the tertiary structure (ΔΔG) and pKa of the catalytic residues H242 and E126. Table S4: Effect of amino acid substitution T216K on the affinity to the zearalenone (ZEA) molecule. Figure S1: The aggregation propensities of *C. rosea* ZHD, the wild-type enzyme and the variants, at different pH values. Figure S2: The solubility of *C. rosea* ZHD, the wild-type enzyme and the variants, at different pH values. Figure S3: Root mean square fluctuation (RMSF) of amino acid residues in the tertiary structure of wild-type *C. rosea* ZHD and the variant T216K in protein dynamics within a coarse-grained protein model.
