# Supplementary material for: The Protein Engineering of Zearalenone Hydrolase Results in a Shift in the pH Optimum of the Relative Activity of the Enzyme
Source: Toxins (Basel). 2024 Dec 13;16(12):540. doi: 10.3390/toxins16120540 (PMC11679840; doi:10.3390/toxins16120540)
Supplement: Supplementary file 1 [file toxins-16-00540-s001.zip › toxins-3315156-supplementary.pdf]

Supplementary material

The Protein Engineering of Zearalenone Hydrolase Results in a Shift in the pH Optimum of the Relative Activity of the Enzyme

Anna Dotsenko, Igor Sinelnikov, Ivan Zorov, Yury Denisenko, Aleksandra Rozhkova and Larisa Shcherbakova

Table S1. Effect of amino acid (aa) substitutions at the positions S103 and G213 on the stability of the tertiary structure (ΔΔG) and pKa of the catalytic residues H242 and E126.

	S103			G213		
**aa residue**	ΔΔG, kcal/mol	ΔpKa(H242)	ΔpKa(E126)	ΔΔG, kcal/mol	ΔpKa(H242)	ΔpKa(E126)
A	0.80508	0.01	-0.01	-0.4896	-0.01	0.08
R	0.48773	-0.05	0.03	-0.50762	-2.19[1]	-2.71
D	1.16059	-0.02	0.02	0.08574	0.33	-1.28
N	0.86929	-0.02	0.02	-0.29387	-0.11	0.32
C	0.35005	0	0	-1.52701	0.19	-0.06
E	0.96132	-0.03	0.03	0.26569	-0.06	0.19
Q	0.68549	-0.03	0.03	-0.09336	-0.05	0.19
G	1.38809	0.02	-0.02	–	–	–
H	0.5498	-0.04	0.04	-0.76735	2.2	-1.92
I	-0.30353	-0.02	0.02	-1.35002	-0.04	0.18
L	-0.3323	-0.02	0.02	-1.33842	-0.11	0.25
K	0.76586	-0.03	0.03	-0.12674	-0.28	-1.63
M	0.15805	-0.02	0.02	-1.08424	-0.04	0.18
F	-0.04165	-0.05	0.05	-1.38029	-0.28	0.42
P	1.06944	-0.01	0.01	-0.58939	-0.03	1.39
S	–	–	–	-0.31105	-0.02	-0.64
T	1.37782	-0.01	0.01	-0.43384	-0.03	-0.56
W	0.24134	-0.08	0.08	-0.94487	-0.24	0.38
Y	0.07349	-0.06	0.06	-1.21234	-0.36	0.36
V	-0.0693	-0.01	0.01	-1.30425	-0.03	0.17

[1] A considerable decrease in the pKa values is indicated in red.

Table S2. Effect of amino acid (aa) substitutions at the positions T216 and F221 on the stability of the tertiary structure (ΔΔG) and pKa of the catalytic residues H242 and E126.

	T216			F221		
**aa residue**	ΔΔG, kcal/mol	ΔpKa(H242)	ΔpKa(E126)	ΔΔG, kcal/mol	ΔpKa(H242)	ΔpKa(E126)
A	0.47994	0.02	0.64	1.39925	0.2	-0.2
R	0.12562	-1.58[1]	-1.46	1.79928	-1.13	-0.99
D	0.90543	-0.01	0.88	1.63569	0.35	-1.7
N	0.23091	-0.01	0.88	1.7989	0.17	-0.03
C	-0.39578	0.01	0.57	0.95177	0.19	-0.19
E	-0.04817	0.47	-0.71	1.49939	0.75	0.05
Q	-0.43622	-0.09	0.50	1.62167	0.16	-0.09
G	0.36835	0.03	0.56	1.81965	0.21	-0.21
H	-0.02002	-0.10	-1.36	1.89911	-0.53	-3.85
I	-0.62222	-0.01	0.88	1.18858	0.17	-0.1
L	-0.65096	-0.01	0.88	0.96811	0.17	-0.03
K	0.32774	-1.00	-0.52	2.27054	-1.12	-1.45
M	-0.84715	-0.08	0.88	1.1729	0.1	-0.1
F	-0.83256	-0.04	0.94	–	–	–
P	0.8301	0.00	0.73	1.72917	0.18	-0.11
S	0.64513	0.01	0.31	1.79858	0.19	-0.12
T	–	–	–	1.44761	0.18	-0.14
W	-0.90579	-0.35	1.22	0.86487	-0.24	0.38
Y	-0.85956	-0.05	0.99	1.21298	-0.15	0.05
V	-0.45947	0.00	0.80	1.04385	0.18	-0.04

[1] See explanation in the caption to Table S1.

Table S3. Effect of amino acid (aa) substitutions at the positions D31 and H125 on the stability of the tertiary structure (ΔΔG) and pKa of the catalytic residues H242 and E126.

	D31			H125		
**aa residue**	ΔΔG, kcal/mol	ΔpKa(H242)	ΔpKa(E126)	ΔΔG, kcal/mol	ΔpKa(H242)	ΔpKa(E126)
A	1.08736	-0.18	-0.03	-0.64955	0.12	1.76
R	1.41119	-1.57[1]	0.03	0.04659	0.06	0.34
D	–	–	–	0.71955	0.2	1.79
N	1.16486	-0.21	0.00	0.06236	0.09	1.79
C	0.59048	0.24	-0.02	-1.3203	0.11	1.77
E	0.53142	-0.22	0.01	0.68818	0.08	1.87
Q	1.03737	-0.22	0.01	-0.00662	0.01	1.51
G	1.69806	-0.10	-0.04	-0.324	0.13	1.75
H	0.87766	-0.30	0.02	–	–	–
I	0.34887	-0.35	0.00	-1.32356	0.09	1.79
L	0.23015	-0.42	0.00	-1.34814	0.02	1.86
K	2.03902	-2.90	0.01	0.57797	0.08	0.5
M	0.38798	-0.35	0.00	-1.21143	0.09	1.79
F	0.21993	-0.66	0.03	-1.34683	-0.08	2.03
P	1.73551	-0.20	-0.01	-0.40019	0.1	1.78
S	1.18087	-0.19	-0.02	-0.59279	0.11	1.77
T	1.2272	-0.27	-0.01	-0.69214	0.1	1.78
W	0.40204	-0.69	0.06	-1.42669	-0.04	2.06
Y	0.36048	-0.74	0.04	-1.3153	-0.09	1.65
V	0.56527	-0.27	-0.01	-1.28553	0.1	1.78

[1] See explanation in the caption to Table S1.

Table S4. Effect of amino acid substitution T216K on the affinity to the zearalenone (ZEA) molecule.

	Wild type		T216K	
**pH**	Affinity, kcal/mol	S102-Oγ – ZEA-carbonyl carbon, Å	Affinity, kcal/mol	S102-Oγ – ZEA-carbonyl carbon, Å
3.0	-9.3	3.4	-9.0[1]	2.4
	-8.8	2.4	-8.3	2.8
4.0	-9.3	3.4	-9.0	2.4
	-8.8	2.4	-8.3	2.8
5.0	-9.3	3.4	-9.0	2.4
	-8.8	2.4	-8.3	2.8
6.0	-9.1	3.4	-9.0	2.4
	-8.8	2.4	-8.0	2.7
7.0	-9.0	3.4	-9.0	2.4
	-8.8	2.4	-7.9	2.9
8.0	-9.0	3.4	-9.0	2.4
	-8.8	2.4	-7.3	3.5
9.0	-9.0	2.4	-9.2	2.4
	-8.8	3.3	-8.1	2.9
10.0	-9.0	2.4	-9.2	2.4
	-8.8	3.4	-8.2	2.8

[1] Numbers in red and black correspond to modes of binding of ZEA at the bottom of the pocket of the active center and remote to the entrance to the pocket, respectively.

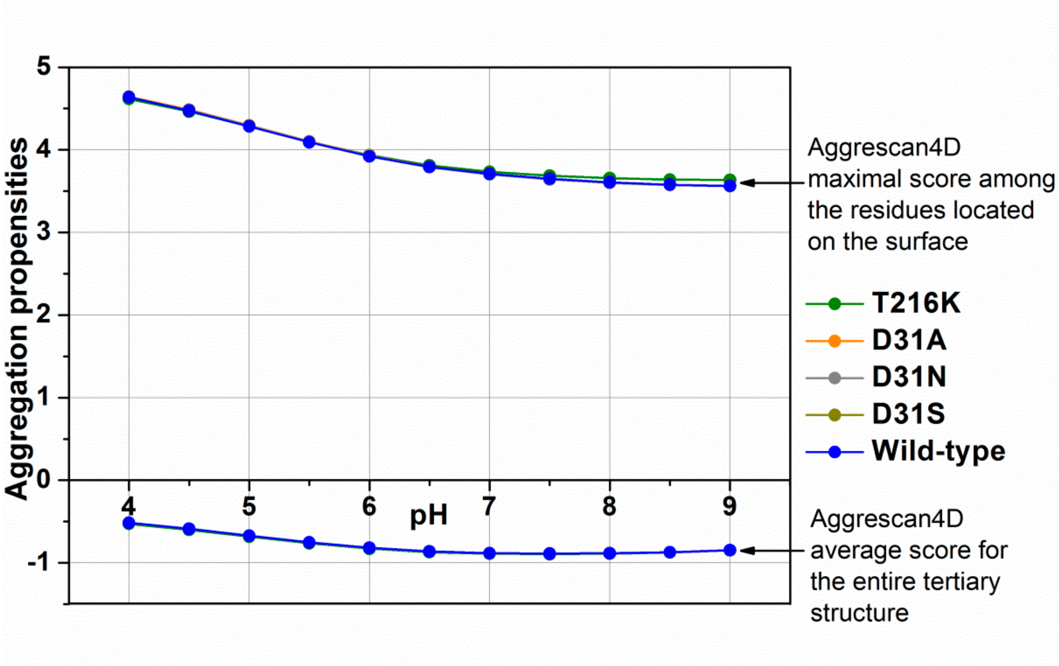

Figure S1. The aggregation propensities of *C. rosea* ZHD, the wild-type enzyme and the variants, at different pH values.

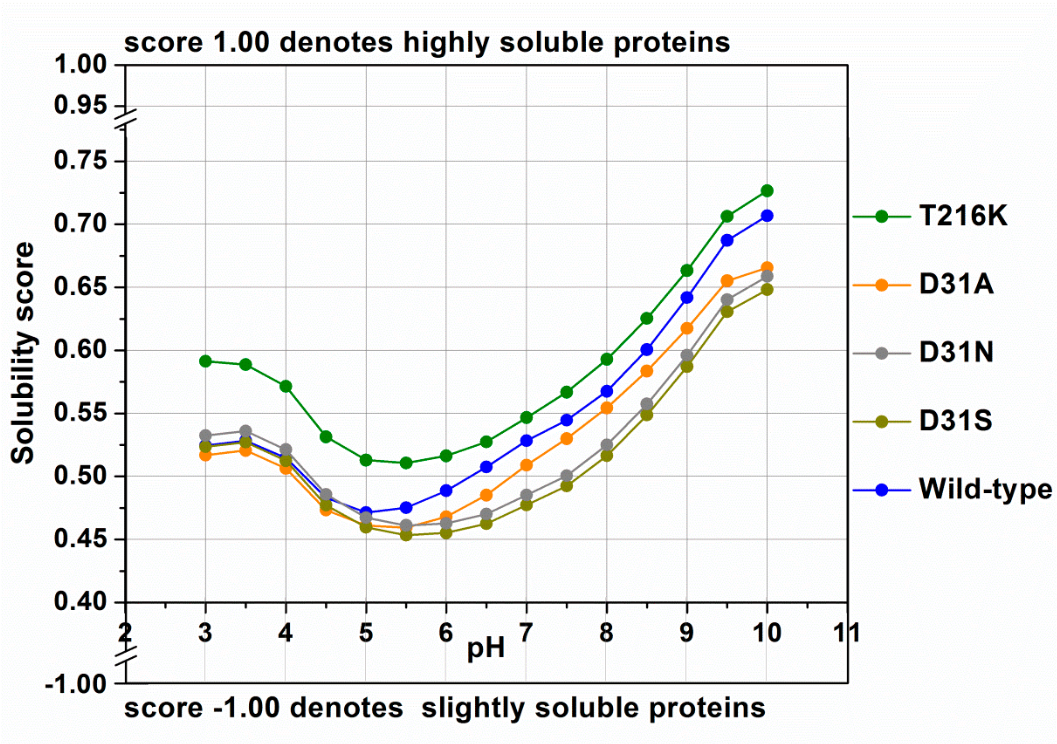

Figure S2. The solubility of *C. rosea* ZHD, the wild-type enzyme and the variants, at different pH values.

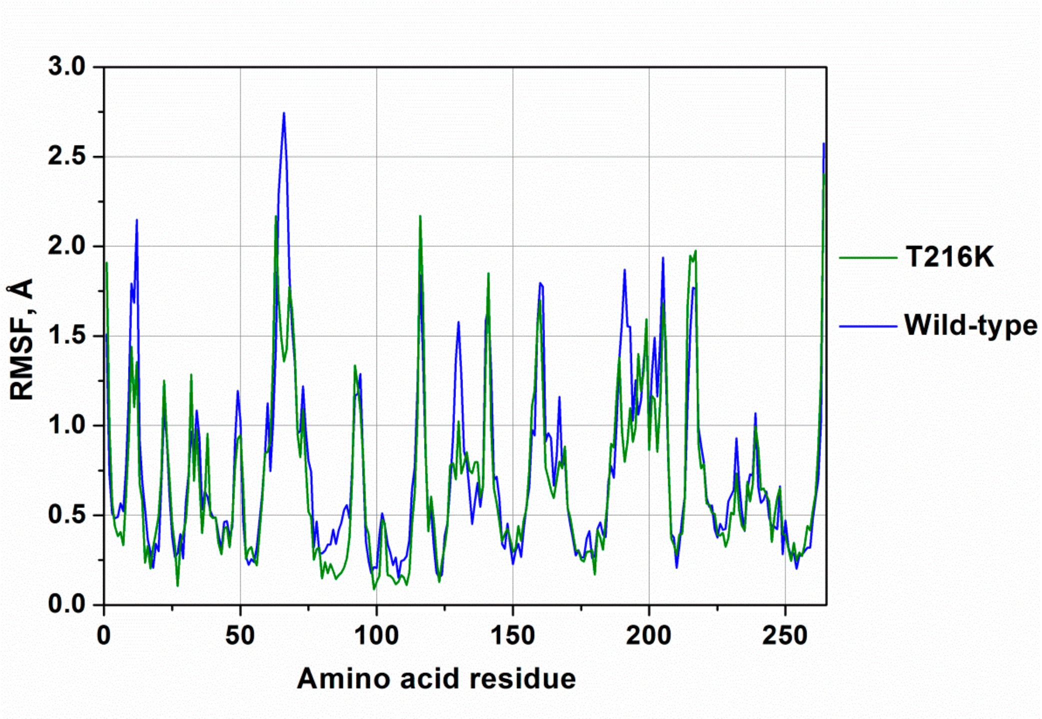

Figure S3. Root mean square fluctuation (RMSF) of amino acid residues in the tertiary structure of wild-type *C. rosea* ZHD and the variant T216K in protein dynamics within a coarse-grained protein model.
